# From paradigm blindness to paradigm shift? An integrative review and critical analysis of the regenerative paradigm

**DOI:** 10.1007/s13280-025-02232-7

**Published:** 2025-09-12

**Authors:** Vanessa Taveras-Dalmau, Susanne Becken, Ross Westoby

**Affiliations:** Griffith University, 170 Kessels Rd, Brisbane, QLD Australia

**Keywords:** Artefact, Neoliberalism, Paradigms, Regenerative, Sustainability, Systems change

## Abstract

Amid growing concerns over sustainable development failures, scholars are exploring the ‘regenerative paradigm’ as a pathway for systemic change; yet, its paradigmatic foundations remain underexamined. Using thematic analysis, we analyse the regenerative knowledge field through an integrative review of 320 cross-disciplinary articles on regenerative approaches, synthesising findings into an interactive Regenerative Paradigm Map with 7 principles, 33 themes, and 253 specific elements. We assess all Map components against an analytical framework of 14 paradigm criteria to evaluate whether regeneration constitutes a paradigm, finding it meets several criteria but not others. We interpret results through the lens of paradigm blindness, interpreted as entrenched worldviews impacting all paradigms. We conclude with a critical reflection on how select Map components may support a shift away from growth-oriented systems and conceptualise the *Tensions of Paradigm Shifts* to discuss blind spots in scholarly interpretations of regeneration, which may contribute to paradigm blindness within the field.

**Supplementary Information:**

The online version contains supplementary material available at 10.1007/s13280-025-02232-7.

## Introduction

Human activity has grown so impactful that we now live in the Anthropocene (Lewis and Maslin 2015). Global environmental change is intensifying, driven by a significant acceleration of material flows beginning in the twenty-first century. Six of nine planetary boundaries, essential for Earth’s stability and resilience, have been transgressed (Richardson et al. 2023), and the chances of limiting warming to 1.5 °C remain slim (IPCC 2023). Sustainable development was proposed as a potential solution, but continues to be criticised. A lingering question is why, despite a 38-fold increase in environmental agencies and laws since 1972, environmental decline continues (Voulvoulis et al. 2022).

Figueroa-Helland and Raghu (2016) identify the breach between Earth’s cyclical metabolism and economic growth-oriented paradigms of Western, Educated, Industrialised, Rich and Democratic (WEIRD) societies (Henrich et al. 2010) as a key driver. They view human–nature relations as embedded in a metabolic interaction that capitalist economies have broken, overshooting Earth’s regenerative capacity. We contend that WEIRD societies exhibit paradigm blindness—so ingrained in growth paradigms that they fail to acknowledge or value alternative ideas (Edelsky 1990). A paradigm shift is needed, and regeneration is emerging as a “radical transformation of economic thought” (Shannon et al. 2022, p. 89).

The regenerative paradigm offers an alternative worldview, emphasising human–nature interdependence (Hes and du Plessis 2015). Despite growing interest, regeneration is criticised for lacking theoretical and practical clarity (Bellato and Pollock 2023). Efforts by Bellato et al. (2022) and Buckton et al. (2023) have advanced understanding, yet gaps remain in analysing regeneration in relation to paradigms: what they are, how they form, become ingrained, and shift. Moreover, most regeneration literature lacks critical scrutiny of its own theoretical foundations. Seemingly, only Paolini et al. (2024) warn of regeneration’s potential co-option, dilution, or exploitation. Building on these critiques, we theorise that regeneration may exhibit early signs of uncritical adoption of simplified heuristics—patterns reminiscent of the same paradigm blindness in growth-oriented systems.

In addition to this lack of reflexivity, scholarship is critiqued for lacking a shared understanding of regeneration’s core components, oversimplifying the concept, and insufficiently integrating diverse knowledge fields (Buckton et al. 2023). There is also a gap between research and implementation, hindering the translation of regenerative principles into actionable strategies (Bellato and Pollock 2023). These limitations create a rationale for exploring regeneration as ‘a paradigm’. To address this, we investigate the question, *What is this thing called the ‘regenerative paradigm’?* inspired by Chalmers’ (1976) seminal book, breaking it into two guiding research objectives.

1. To map out the components comprising the regenerative knowledge field.
2. To assess the components of the regenerative knowledge field against established paradigm criteria to determine whether regeneration, as interpreted in current scholarship, constitutes a paradigm.

We conduct an integrative review of 320 cross-disciplinary regeneration articles, using inductive and deductive thematic analysis. Prioritising depth over breadth, we deepen our focus on tourism—a complex microcosm of human–environment interaction with significant transferability to other systems—while maintaining a broad analytical lens to ensure insights are relevant to other fields. Findings are synthesised into an interactive Regenerative Paradigm Map detailing regeneration’s ‘what, why, and how’, which we assess against 14 paradigm criteria to evaluate whether regeneration constitutes a paradigm. We interpret our findings through a conceptual lens of paradigm blindness and what we conceptualise as the *Tensions of Paradigm Shifts.* The study provides a theory-grounded, action-oriented foundation for exploring paradigm blindness in WEIRD societies while identifying blind spots and tensions within regeneration scholarship that may reflect, or risk contributing to, paradigm blindness within the field.

## Theoretical framework

### Theoretical foundation of paradigms

Individuals interpret the world through socially constructed mental models or lenses (Meadows 2009), formed unconsciously through life experiences and social interactions. Acting as cognitive, affective, and perceptual maps, these lenses help individuals make sense of the world and align with societal norms, collective ideas, and shared culture (Olsen et al. 1992). They are so fundamental to cognitive and perceptual abilities that people seldom recognise or question them (Meadows 2009). *Shared* mental lenses underpin dominating worldviews, which are reinforced by prevailing values and cultural, political, and economic structures, influencing what is considered acceptable, rational, or desirable.

A worldview is “a cosmological framing of how things work” (Sanford 2019, p. 14), comprising *beliefs*, *belief systems*, and *values.* Beliefs are ideas held as ‘true’ despite possible counterevidence. A belief system consists of interconnected ideas shared by a community. While beliefs concern what we think *is,* values represent what *should be*, reflecting desirable or undesirable outcomes and guiding prioritisation, decision-making and action (Olsen et al. 1992). Although worldviews are often equated with paradigms, they are distinct, playing different roles in systems transformation.

Thomas Kuhn (1962) defined paradigms as “the entire constellation of beliefs, values, techniques, and so on shared by members of a given community” (p. 175). Like ‘mini-worldviews’, a society may contain many paradigms, each with its own beliefs, belief systems, and values, all collectively influenced by the dominant (or competing dominant) worldview(s) (Olsen et al. 1992). Although a paradigm is composed of diverse attributes not strictly defined in the literature, Masterman (1970) identified 21 interpretations of Kuhn’s definition, categorising paradigms as “metaphysical”, “sociological”, and “construct”, each associated with specific criteria.

According to Kuhn (1962), paradigms set the boundaries and norms of scientific inquiry, acting as a “puzzle-solving” map guiding scientific observation and experimentation. Though initially confined to science, paradigms were later recognised to shape broader societal worldviews—what Dunlap and Van Liere (1978) termed “Dominant Social Paradigms”. Paradigms form unconsciously, with rules defining boundaries and norms (Prince 1994). As societies grow more complex, additional rules solidify the paradigm, shaping perceptions of reality and creating an inescapable metaphorical ‘box’ (Prince 1994). Ultimately, individuals unconsciously accept the paradigm as ‘the way things are’. If such acceptance remains uncritical and unquestioned, paradigm blindness emerges.

### Exploring paradigm blindness

Edelsky (1990) coined the term paradigm blindness, referring to researchers’ inability to recognise they are so ingrained in their paradigm they cannot see, appreciate, or acknowledge the legitimacy of ideas outside their paradigm. We extend this concept to describe how individuals, organisations, and societies unconsciously accept paradigms as unquestionable truths. This can lead people to assume they speak for others, imposing their own agenda while expecting others to cooperate in its pursuit (Edelsky 1990). Paradigm blindness, therefore, obstructs the ability to perceive or accept challenges to dominant worldviews, which, in turn, impacts learning, innovation, policy-making, and broader systems change (Fischbacher-Smith 2012).

Paradigm blindness forms when a paradigm becomes deeply entrenched, resembling an ideology—an argument that explains, justifies, or legitimates actions or goals (Kinlock 1981). Ideologies are typically formulated to further the power, privileges, actions, and goals of specific actors (Olsen et al. 1992). This applies to economic growth, which Barry (2018, p. 1) calls “an ideology…of the capitalist state…that serves the interests of a specific class or elite, rather than…the interests of a majority in society”. McMaster (2015) also argues that, as doctrine, *homo economicus* resembles religious fundamentalism and is hence a source of paradigm blindness.

Bouchaud (2008) posits that “economics needs a scientific revolution”, evoking Kuhn’s (1962) paradigm shift and speaking to the need for transforming the fundamental assumptions and methods guiding economics. Growing criticism of the pursuit of infinite economic growth signals the early stages of such a shift, as current anomalies, such as widening social disparities, undermine confidence in growth-oriented paradigms (Schmelzer 2015). However, paradigm shifts cannot occur unless a new paradigm emerges that offers different standards and a legitimate mandate (Chalmers 1976).

### The paradigm shift cycle

**Fig. 1:**
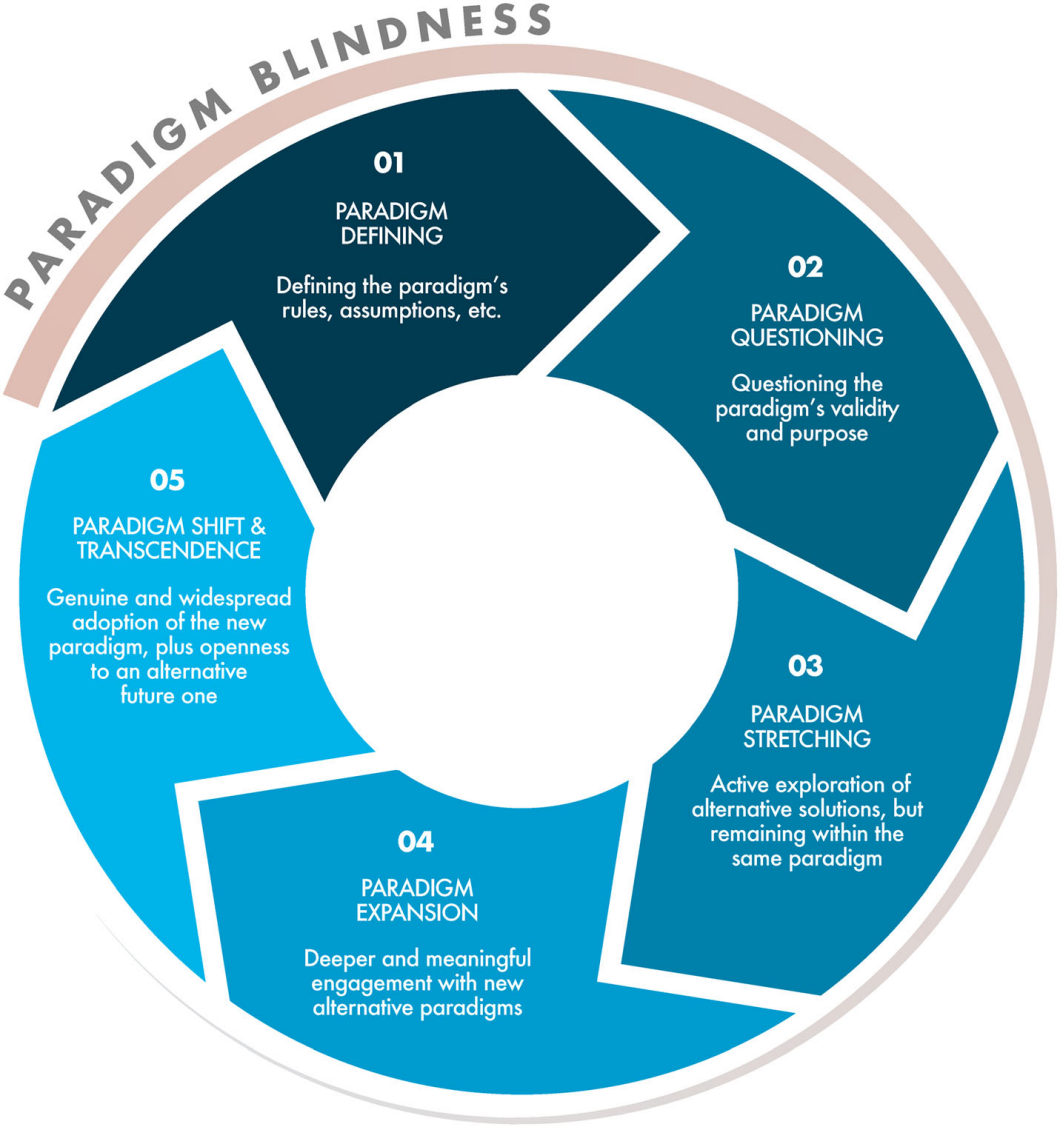
The Paradigm Shift Cycle. Inspired by Prince (1993) and (1994)

Paradigm shifts typically follow a metaphorical five-stage cycle (Fig. 1), beginning with the definition of the paradigm (Stage 1). When the paradigm solidifies into unquestioned beliefs, paradigm blindness develops. As knowledge advances, anomalies emerge that cannot be resolved using the paradigm’s rules. Initial reactions often deny and minimise anomalies (Prince 1994). However, when such efforts fail, individuals begin ‘removing the blindfolds’ to question the paradigm’s rules, beliefs, and assumptions, assessing their validity, purpose, and relevance (Prince 1994) (Stage 2). As they do so, they might propose alternative solutions—ones that remain aligned with the dominant paradigm (Stage 3).

As these fail, deeper engagement with an alternative paradigm begins (Stage 4). Ultimately, the failures of the dominant paradigm become undeniable, prompting a shift towards an alternative one that is better suited to evolving knowledge (Stage 5) (Prince 1993). However, paradigms are not static; if adopted uncritically, they become entrenched too, initiating a new cycle that creates paradigm blindness once again. This cycle reveals a central paradox of paradigm shifts: while new paradigms emerge to challenge dominant systems, they too risk becoming an ideology, replicating the very paradigm blindness of the ‘old’ one.

### Evolution of paradigms influencing human–nature relationships

Environmental ethics literature often highlights two dominant beliefs shaping society’s relationship to nature: anthropocentrism and ecocentrism, both rooted in a human–nature dualism influencing modern environmental thinking (Purser et al. 1995). Anthropocentrism holds that higher cognition, language, and the ability to reason and use advanced tools position humans at the centre of the moral universe (Goralnik and Nelson 2012), judging actions towards nature solely by their impact on humans (Eckersley 1992). Consequently, anthropocentrism expects nature to serve humans, with technology resolving any arising issues (Lundmark 2007).

This faith in progress and prosperity is often associated with Western paradigms in WEIRD societies, the focus of this study. The Enlightenment positioned “scientific inquiry” as the dominant pursuit, driven by “Western man” (Berry 1978, p. 189), fostering a reductionist and exploitative attitude towards the environment, leading to its “despiritualisation”, and removing ‘God’ and the divine from nature (Palamos 2016). Subsequently, several paradigms emerged separating humans from nature (see Supplementary Materials, Appendix S1). While some paradigms reference sustainability, they reflect an anthropocentric interpretation aligned with Naess’ (1973) “shallow ecology”, rather than the normative environmental philosophy of “deep ecology”.

Contrasting anthropocentrism, ecocentrism emphasises that all life has value. While attributing value is inherently a human act of judgement, ecocentrism fosters an ethic of respect for nature’s complexity, worth, and interdependence (Lundmark 2007). Before Western environmental ethics conceptualised these terms, Indigenous spiritualities reflected an ecocentric worldview (Waters 2024). In parallel, DeWeese (2023) shows that theocentric traditions also emphasised “creation care”, addressing environmental preservation, conservation, and restoration. Modern ecocentrism builds upon these diverse perspectives, contributing new paradigms preceding regeneration that redefine human–nature relations (see Supplementary Materials, Appendix S1).

Yet, current systems remain rooted in neoliberal ideology, prioritising economic growth over social-ecological health. Neoliberalism has become “an asphyxiating monolith…from which our critical thinking cannot escape”. Increasingly, scholars view neoliberalism as unfit for purpose, calling for a ‘new economics’ (Buckton et al. 2024; Kenter et al. 2025) that shifts neoliberalism’s underlying beliefs: competitiveness, deregulation, efficiency, free markets, profit, consumption, capitalism, globalisation, individualism, and growth (Tribe et al. 2015). We argue that neoliberalism’s fixation on “growthmanship” (Schmelzer 2015) reflects a paradigm blindness that WEIRD societies must overcome to transform economies in service to life (Lovins et al. 2018). Regeneration is being proposed to spark such transformation.

### Regeneration as an alternative paradigm?

Regeneration, the core characteristic of living systems, seeks to address system degradation by restoring their capacity to function at optimal health (Sanford 2019). Regeneration is positioned as a ‘new’ paradigm, yet gaps persist in understanding it through a paradigmatic analysis, particularly regarding how paradigm blindness may shape its theoretical framing. This study addresses these gaps by analysing regeneration against a backdrop of what paradigms are and how they form and shift. We map regeneration’s theoretical components, assess its alignment with paradigm criteria, and critically examine blind spots that hinder conceptual and practical clarity.

We interpret our findings through the lens of paradigm blindness, discussing tensions that may signal its presence within current regenerative discourse or that risk creating it in the future. Exploring these dynamics is crucial, as uncritical acceptance of the paradigm’s assumptions can undermine its transformative potential. Without reflexivity and a willingness to confront conceptual and practical tensions, regeneration risks becoming an ideology. Left unchecked, this paradigm blindness could reduce regeneration to a commodified narrative, one that inadvertently replicates the same paradigm blindness it seeks to replace in growth-oriented systems.

## MATERIALS AND METHODS

### Researcher positionality

Positionality refers to a researcher’s worldview and stance when conducting research (Yip 2024). We share the regenerative vision that defective paradigms contribute to systemic social-ecological failures. However, we critically examine regeneration, recognising that accepting it unquestioningly risks perpetuating new forms of paradigm blindness. Moreover, as an islander from the Global South residing in a Western country and two Western academics, we acknowledge that our WEIRD cultural lenses influence how we interpret findings and the so-called paradigm shift, posing the risk of displaying our own paradigm blindness.

We are also cognisant of the tension between applying scientific methods and the regenerative paradigm’s philosophy of potentially challenging such methods. To mitigate our paradigm blindness as scientists, we drew on diverse cross-disciplinary and grey literature beyond our immediate expertise. Our findings honour the paradigm’s diversity by preserving even subtle insights. We also actively engaged in metacognition and critical reflection on both existing scholarship and our own biases. In doing so, we aim to spark debate, encouraging others to “see our worldview rather than seeing with our worldview” (Sterling 2011, p. 23).

### Integrative review approach and analytical framework

We combined an integrative review with inductive and deductive thematic analysis. Literature was collated using the Preferred Reporting Items for Systematic Reviews and Meta-Analyses (PRISMA) method (Page et al. 2021). Following Lubbe et al. (2020), we extracted, synthesised and critiqued data to generate a new model—a Map—rather than simply reporting on previous research. Inductive analysis identified the regenerative knowledge field components, while deductive analysis addressed research objective number two, guided by an analytical framework of 14 paradigm-defining criteria (Table 1), to assess whether regeneration constitutes a paradigm. Criteria with substantial overlap were merged to balance analytical clarity with appropriate granularity.

**Table 1:** Analytical framework of paradigm criteria, sourced from Anand et al. (2020), Chalmers (1976), Kuhn (1962), Meadows (1999), Masterman (1970), Olsen et al. (1992), Prince (1994), and Tribe et al. (2015)

Paradigm criteria	Description
Tenets	Fundamental principles that govern perception and action within a paradigm, providing an overarching structure for how the paradigm operates and evolves
Rules, standards, and judicial decisions	Formalised or preconceived norms that paradigm holders adhere to, with their consensus acting as prerequisites for how the paradigm operates, defining boundaries, procedures, and acceptable practices
Epistemology and knowledge	Concerns the structure, nature, and evolution of knowledge within a paradigm. This includes what we accept as ‘knowledge’, how it is vindicated by evidence and how it shifts and grows
Beliefs and assumptions	Relates to the cultural and ideological underpinnings of a paradigm that dictate the specific ideas individuals have about aspects of life that they are convinced are true regardless of contrary evidence. These are often unspoken or implicit, but profoundly shape the paradigm’s development and application
Ontology and worldviews	Refers to the foundational perspectives and assumptions about reality that underpin a paradigm, including subconscious ways of seeing and what we accept as ‘real’
Political institutions	Structures of governance, power, and decision-making that influence and regulate a paradigm. They shape its application through policy and societal organisation
Tools, models or instruments	Conceptual, instrumental, or analytical tools supplied by the paradigm itself, which are employed consciously or unconsciously to apply or operationalise the paradigm, evolving only when they no longer address anomalies within the paradigm
Language	Refers to the terminology and shared system of communication that shapes methodologies, unites practitioners, expresses the paradigm’s ideas, and evolves alongside theoretical and methodological shifts to maintain a common base for inquiry and collaboration
Methods, techniques, and processes	General methodological prescriptions or standard ways of applying the paradigm’s rules, acting as instrumental techniques that bring those rules into the real world to achieve its objectives, providing actionable pathways for implementation
Frameworks	Structured conceptual systems that guide understanding, observation, and action within paradigms, framing scientific progress and the evolution of knowledge
Intuitions	Intuitions are shared, often tacitly communicated instincts and insights within a paradigm, shaping its unspoken foundations beyond its formalised rules. These often emerge from personal or collective experience and can shape decision-making
Values	The shared ethical, moral, and social principles that underpin and justify the paradigm, expressing collective judgments about what is desirable and guiding actions by defining how things ought to be in alignment with the paradigm’s beliefs, thus influencing broader paradigm goals
Metaphysical speculation	The foundational assumptions and philosophical interpretations that shape how phenomena are described and understood, influencing ideas about existence and reality. Metaphysical speculation acknowledges that paradigms, while unifying perspectives, never fully explain all observable facts
Theories	Structured body of knowledge derived from scientific observations and experiments that serves as a foundational framework within a paradigm to explain phenomena and make predictions, bridging empirical evidence with broader scientific understanding

### Data collection

In April 2022, the lead author conducted a keyword-informed search in ProQuest, SAGE, Scopus, ResearchGate, and Web of Science using the terms “regenerative economy”, “regenerative development”, “regenerative design”, “regenerative sustainability”, “regenerative futures”, and “regenerative tourism”. “Regenerative agriculture” was excluded due to its tendency to focus on agricultural practices rather than socio-economic-ecological interactions. The inclusion criteria included English or Spanish books, chapters, and journal articles published between 2007 and 2023 with search terms in their titles, abstracts, or keywords.

Initial searches in Scopus, Web of Science, and ProQuest generated an unmanageable volume of records, many of which were irrelevant to the regenerative knowledge field. To improve the relevance of search results, we removed subject areas unrelated to our study, such as computer science, molecular biology, medicine, gene expression, among others, excluding literature from those fields. Instead, we iteratively refined the search terms and inclusion criteria to ensure that the final sample only contained literature relevant to regeneration in social, economic, and ecological contexts.

To better capture the regenerative paradigm’s emergence in tourism, we expanded the inclusion criteria for regenerative tourism only. This included adding “regenerative travel” and “turismo regenerativo” as terms and broadening the range of databases and publication types to include Google Scholar, Academia.edu, Medium, LinkedIn, reports, theses, conference proceedings/papers, tourism expert blog articles, webinars, and podcasts. These additional sources were used exclusively for tourism-related literature. Another search was conducted in April 2024, using all keywords to capture newly published literature within and beyond the tourism sector.

**Fig. 2:**
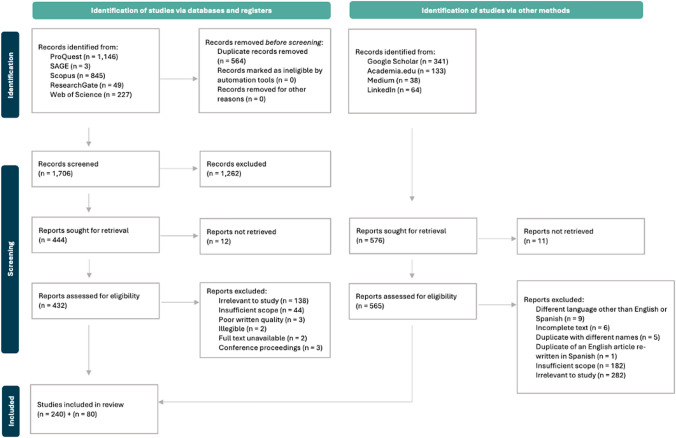
PRISMA flow diagram detailing the systematic process undertaken to collate relevant articles. Reports excluded were those that had no relevance to the study, contained insufficient scope (e.g. where the search terms appeared 1–2 times and the article did not engage meaningfully with regenerative concepts), were poorly written or were not legible, whose full text was not available or that constituted conference proceedings but were incorrectly categorised as journal articles in academic databases. Appendix S3 in the supplementary materials includes a PRISMA checklist

Figure 2 tracks the ‘flow’ of information through the PRISMA protocol (Lubbe et al. 2020). Databases and registers rendered 2270 records, exported into Excel with abstracts. After removing duplicates, 1706 records remained. We screened all abstracts and excluded 1262 records without abstracts, not meeting inclusion criteria, in different languages, or containing unrelated keywords, leaving 444 for retrieval, of which 12 were not retrievable. We assessed the full texts of 432 articles, excluding 192 for several reasons (Fig. 2). An additional 576 records were identified via other methods (Fig. 2), with the same process repeated for these articles. Overall, 320 articles were included in the final review.

### Data analysis

Guided by Fereday and Muir-Cochrane (2006) and Williams and Moser (2019), we inductively coded all articles in NVivo 14. Open coding identified main concepts, generating an extensive list of codes. Peer debriefing and a research journal with notes, diagrams, and insights supported the analysis (Nowell et al. 2017). Using axial coding, we refined open codes into categories, moving iteratively between open and axial coding to create broad thematic groupings. Researcher triangulation mitigated potential biases (Helen and Roberta 2019). Selective coding organised the data further, with peer debriefing vetting and refining codes.

**Fig. 3:**
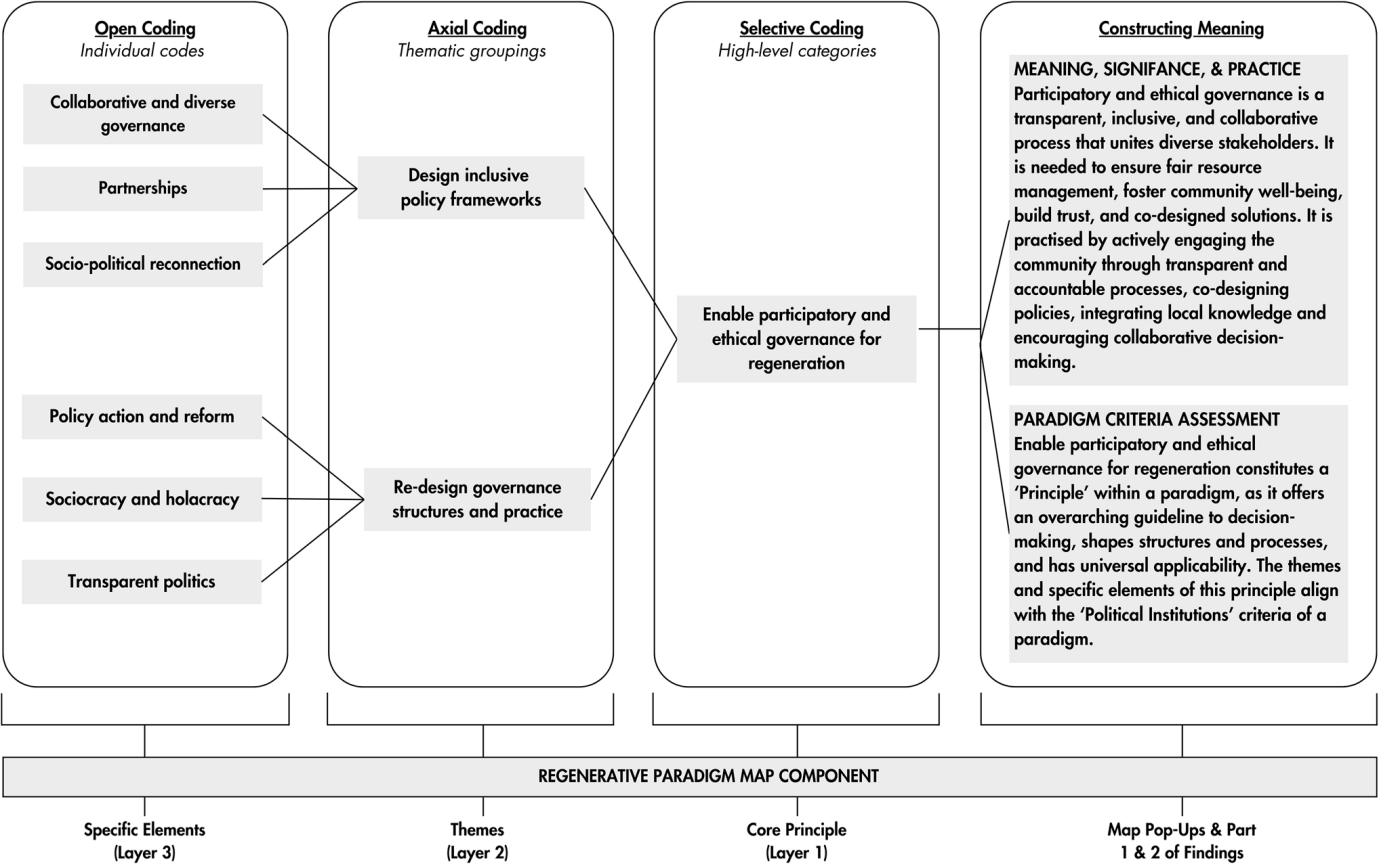
Open, axial, and selective coding process using an example from the Map. Codes were translated into layers and informed the meaning, significance, practice analysis, and paradigm criteria assessment

Final open, axial, and selective codes informed the three layers of the Regenerative Paradigm Map (Fig. 3). Using Flourish (Canva 2025), we created a data visualisation, then reviewed each component to explore its meaning, significance, and practical implications (i.e. constructing meaning, Fig. 3). We created pop-ups for all specific elements—small interactive boxes appearing when a user hovers over them—to explain their ‘what, why, and how’, using Artificial Intelligence (ChatGPT 4) to extract the information from the data; all outputs were reviewed manually.

We assessed all Map components against the analytical framework (Table 1), further constructing meaning (Fig. 3). We then applied a critical interpretive lens grounded in the concept of paradigm blindness to identify theoretical gaps, ambiguities, or tensions in existing scholarship. This lens informed a critical reflection that underpinned our discussion. Drawing on Elliot and Timulak’s (2005) notion of “critical challenge” (p. 152), we approached the findings with self-reflection and scepticism, probing their implications for attributes that may reflect paradigm blindness within existing regeneration scholarship.

### Limitations

While efforts were made to ensure comprehensive coverage, some articles, particularly from non-academic databases with limited search functionality, may have been missed. These sources were not the study’s primary focus, but captured emerging regenerative practices. Academic database searches were extensive, supported by alerts to track new publications. Given the expanded inclusion criteria for tourism scholarship, approximately 50% of articles were tourism-related. To assess bias, we tested the impact of removing grey tourism-related literature on coding, resulting in only 12 deleted codes. Given the minimal impact, we retained the original sample. Finally, assessing the paradigm criteria involved a degree of subjectivity and interpretive judgement, informed by our interdisciplinary background and our engagement with paradigm scholarship.

## RESULTS

**Fig. 4:**
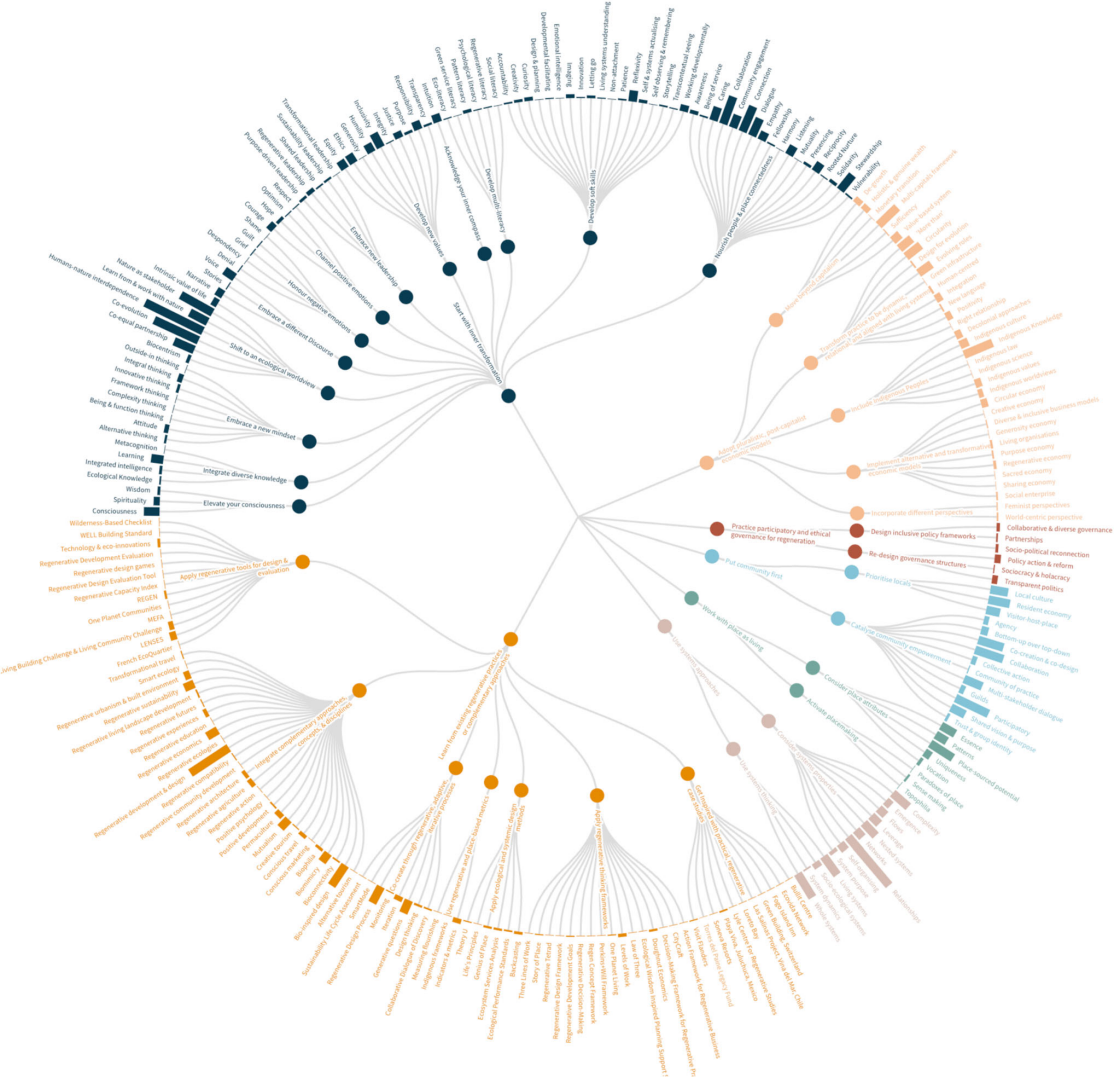
The complete Regenerative Paradigm Map: The innermost layer represents the seven core principles. The second layer represents the 33 themes. The outermost layer represents the 253 specific elements. Readers can visit the online interactive map version here: https://bit.ly/RegenerativeParadigmMap

**Fig. 5:**
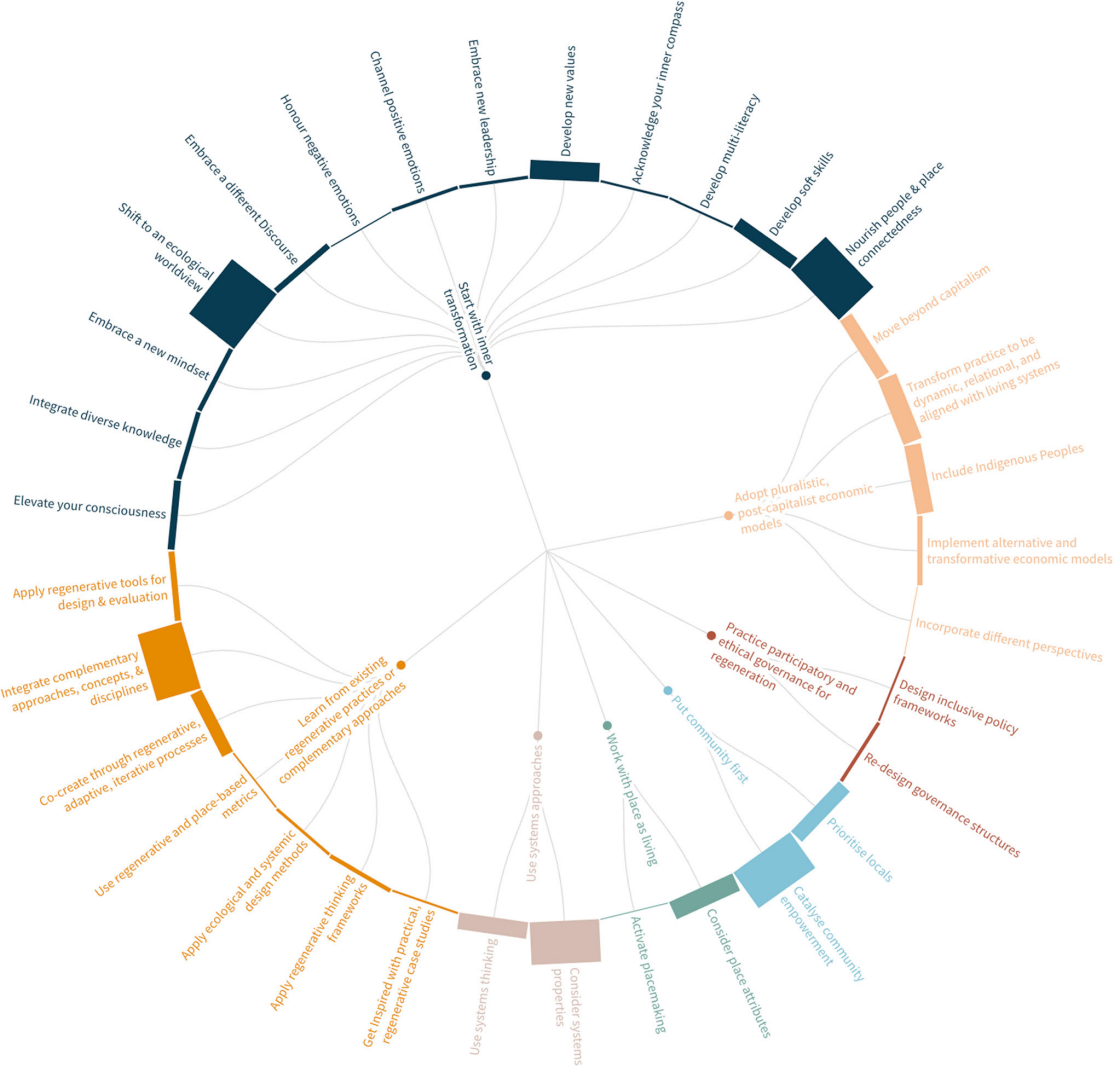
Core principles (layer 1; inner) and themes (layer 2; outer) of the Regenerative Paradigm Map

In accordance with the research objectives, findings are presented in two parts. Part 1 outlines the Regenerative Paradigm Map (Fig. 4), featuring 7 core principles, 33 themes, and 253 specific elements, the latter representing concepts or practices that further conceptualise or operationalise regeneration. Figure 5 provides a more detailed view of core principles and themes. All Map components are italicised in the text for clarity. Due to word constraints, specific elements are explained in interactive pop-ups in the online Map. Part 2 of the findings assesses whether regeneration meets the paradigm criteria (Table 1), using evidence from the Map.

### Regenerative paradigm map

#### Core principle 1: start with inner transformation

The first core principle involves the ‘inner world’, reflected in 86% of articles, reinforcing the need for complementing external solutions with inner transformation, referring to changes in worldviews, consciousness, beliefs, etc. (Mathisen et al. 2022). Taveras-Dalmau (2024) conceptualises this transformation as inner regenerative development. Authors argue that creating regenerative futures will be impossible without integrating inner/outer dimensions of sustainability that challenge dominant paradigms separating humans from nature (Herranz-Pascual et al. 2023). The reviewed literature suggests *inner transformation* requires ‘inner work’ across several inner domains, illustrated in the 13 themes in the outermost layer of Fig. 4.

#### Core principle 2: adopt pluralistic, post-capitalist economic models

The second principle, identified in 66% of articles, underscores the need to move beyond growth-oriented systems that undermine long-term human and ecological well-being. These systems contribute to biodiversity loss, inequality, and cultural disruption (Izquierdo Gascon and Gil 2023). Yet, such impacts are often underreported, as success remains measured mainly in financial capital rather than social, human, and natural capital. The literature indicates that *Principle 2* requires *moving beyond capitalism*, *transforming practice to be dynamic, relational, and aligned with living systems*, *implementing alternative and transformative economic models*, *incorporating different perspectives*, and *including Indigenous Peoples*.

#### Core principle 3: practise participatory and ethical governance for regeneration

This principle, identified in only 10% of articles, tackles the challenge of “finding effective and ethical ways of decision-making” (Dietz 2020, p. 93) to manage resources for the well-being of all (Sala Benites et al. 2023). ‘Good’ governance addresses challenges in complex economic systems, fostering collaboration between communities, government, and industry, creating spaces for alternative ideas to be tabled, negotiated, and reconciled (Teruel 2018). According to the literature, it entails *[re]designing governance structures* and *inclusive policy frameworks* to enhance participation, adopting *collaborative and diverse governance*, *reforming policy*, and *reconnecting environmental policy with socio-political dimensions* (Shannon et al. 2022).

#### Core principle 4: put community first

Identified in 63% of articles, this principle prioritises local communities and their well-being above the interests of external actors and narrow economic gains (Tomassini and Cavagnaro 2022). The principle is presented as a central premise of regeneration because of communities’ lived experiences and in-depth knowledge about their place’s historical, social, and ecological circumstances, vital for creating regenerative systems (Axinte et al. 2019). Various articles indicate that this principle requires *prioritising locals* and *catalysing community empowerment*, supported by factors like respecting *local culture* and giving *agency* to locals to ensure development is *co-created* using a *bottom-up approach* (Buckton et al. 2023).

#### Core principle 5: work with place as living

*Work with place as living*, identified in 46% of articles, recognises each place as a unique, complex, and adaptive living system with distinct regional, biological and cultural characteristics (Darcin 2020). The literature highlights that WEIRD paradigms prioritise universal theories or models that commodify and homogenise place, “ironing out the diversity that makes place unique, interesting, and resilient” (Mang et al. 2016, p. 56). Hence, regenerative approaches oppose standardised methods typical of WEIRD paradigms, instead advocating for working in ways that foster connection with place by understanding its *story*, *patterns*, and *potential* and then designing projects in alignment with such factors.

#### Core principle 6: use systems approaches

This core principle appeared in 68% of articles, highlighting the need to shift from reductionist towards holistic ways of thinking (Bruno 2019). Hes and du Plessis (2015) position this as a central premise of the *ecological worldview*, helping practitioners recognise that the world is complex and works as a system of nested wholes rather than a mere collection of parts, thus fostering an understanding of the interconnections and relationships inherent in the systems they work in. The literature suggests the principle requires *considering systems properties*, like *emergence*, *complexity*, *flows*, *leverage*, etc., and *employing living systems thinking methodologies*.

#### Core principle 7: learn from existing regenerative practices or complementary approaches

Numerous methods, frameworks, and tools contribute to the practice of regeneration, hence Principle 7*,* appearing in 59% of articles. Ultimately, we found that regeneration is a ‘rediscovery’ of an old view (du Plessis 2012), encompassing many existing practices that support its conceptualisation or operationalisation. Authors suggest this principle entails *applying regenerative thinking frameworks* and *regenerative tools for design and evaluation*, *co-creating through regenerative, adaptive, iterative processes*, *using regenerative and place-based metrics*, *applying ecological and systemic design methods, integrating complementary approaches, concepts, and disciplines* and *getting inspired with practical, regenerative case studies.*

### Assessment against paradigm criteria

In this section, the findings articulated in the Map are assessed against 14 paradigm criteria (Table 1) to evaluate whether regeneration meets the characteristics of a paradigm. Alongside this assessment, we offer interpretive critiques of theoretical gaps in the literature informed by the concept of paradigm blindness, the Paradigm Shift Cycle (Fig. 1), and areas that were underdeveloped in relation to each criterion—issues that may affect how regeneration is conceptualised. Appendix S2 in the Supplementary Material nests all Map components under each criterion.

#### Tenets

This criterion is reflected in the literature, with the Map’s seven core principles framing regeneration, offering direction for how individuals, communities, and entire systems operate within the paradigm. Combined, the core principles reflect most paradigm criteria underpinning regeneration. They are an invitation to align inner change with outer change, replace linear approaches with alternative models, create governance systems rooted in transparency and collaboration, prioritise communities, work with places as living, dynamic systems, employ living systems approaches that consider interconnectedness, and build on existing practices.

#### Rules, standards, and judicial decisions

This criterion is partially met through the theme of *prioritising locals.* The specific elements of *collaboration*, *community engagement*, *local culture*, *green infrastructure*, *degrowth,* and *participatory approaches* broadly constitute the regenerative paradigm’s rules, standards, and judicial decisions. They emphasise *prioritising local communities* in decision-making, shifting from top-down governance to *participatory* systems where locals are *co-creators* of solutions. Critically, though, as positioned in the literature, the regenerative paradigm lacks clearly articulated rules, standards, and judicial decisions, limiting clarity around its paradigmatic foundations.

Legally binding mechanisms are also absent, with the words ‘judicial’ and ‘Indigenous law’ appearing in only two articles. One references judicial processes that denied Aboriginal Australians sovereign rights to Country (Wooltorton et al. 2022). The other describes Indigenous law but with no connection to legal mechanisms (Liaros 2020). Without alignment with existing legal frameworks and the development of robust accountability mechanisms, integrating the regenerative paradigm into governance frameworks remains unclear.

#### Epistemology and knowledge

Regeneration draws on multiple epistemologies, meeting this criterion by *integrating diverse knowledge, practices, and perspectives* alongside *feminist* and *world-centric perspectives*. It advocates for *shifting mindsets*, *embracing alternative thinking*, and *developing new skills* and *multiple forms of literacy*, like *eco, social, pattern*, and *regenerative literacy* (Hes and du Plessis 2015; Becken and Coghlan 2022). Yet, despite advocating for diverse epistemologies, the literature is predominantly authored by Western academics and communicated through Western science formats.

#### Beliefs, belief systems, and assumptions

This criterion is met by the literature positioning the regenerative paradigm as a shift from anthropocentric beliefs towards *human–nature interdependence*, *solidarity*, *co-evolution*, *reciprocity*, and *value-based* beliefs *nourishing people and place connectedness.* These beliefs, central to global flourishing, are contrasted with WEIRD paradigms, but lack clearer articulation and empirical testing. Moreover, the literature tends to emphasise ‘place’, without seldom engaging with the tension between place-based initiatives and the global systems within which they operate.

In a tourism context, for example, scholars advocate for tourism development that highlights the unique aspects of a destination (Andersson 2022). Yet, issues of scale concerning dependencies on global tourism flows, such as cruise ships, long-haul travel, or capitalist economic structures that facilitate [regenerative] tourism, are overlooked. Finally, many specific elements risk romanticising a future that may lack acceptance in certain cultures. Exploring how beliefs form, evolve and how cognitive biases reinforce internalised views (Grayling 2011) is crucial, yet remains absent in regeneration scholarship. Addressing these complex psychological processes is critical to avoid replicating paradigm blindness.

#### Ontology and worldviews

The literature meets this criterion, advocating a shift in WEIRD societies’ mechanistic worldviews towards *ecological worldviews* informed by *interconnectedness*, *decolonial approaches*, *Indigenous knowledge*, and *systems thinking*. Specific elements like *essence*, *place-sourced potential*, and *complexity* reflect this shift. However, the literature often assumes Western and Indigenous worldviews can seamlessly coexist despite operating under fundamentally different beliefs and values. Little attention is given to “building a meaningful bridge” through decolonial approaches (Datta and Starlight 2024, p. 1), nor is there broad consideration of other worldviews. For example, only seven articles provide a deep engagement with African cultures.

#### Political institutions

This criterion appears in the literature as a call for moving away from centralised, top-down governance models and towards more *inclusive*, *collaborative,* and *transparent* ones (Axinte et al. 2019). Themes of *inclusive policy frameworks*, *re-designing governance structures*, and *including Indigenous Peoples* reflect this approach. In parallel, *sociocracy and holacracy* offer *collaborative and diverse governance* models that foster *partnerships* with diverse actors and promote *agency* within communities (Buckton et al. 2023). However, the literature lacks clarity on how to integrate collaborative governance frameworks into existing political institutions and provides limited discussion on creating mechanisms to *include Indigenous Peoples* on their terms.

#### Tools, models, or instruments

The literature discusses many tools, models, or instruments to operationalise the regenerative paradigm. For example, the principle *learn from existing regenerative practices or complementary approaches* and the theme *apply regenerative tools for design and evaluation,* introduce tools like *LENSES* and *Living Building Challenge* (Hes and du Plessis 2015), alongside *case studies*. Nonetheless, documentation on tool effectiveness remains limited. While some are applied in specific *case studies*, their context-dependency hinders replicability, posing challenges for broader implementation. However, the literature emphasises that tailoring approaches to specific contexts is essential.

#### Language

This criterion is reflected in the literature through the theme of *embracing a new discourse*, supported by *narrative*, *stories*, *voice*, and *new language*. It highlights the need for crafting a ‘new story’ that promotes flourishing within ecological limits, includes unrepresented *voices*, and delivers well-being for all (Lovins et al. 2018). While powerful, the discourse reflects aspirational ideals, sometimes romanticising autopic transition without fully considering systemic complexities. Moreover, the language used often remains technical, abstract, or occasionally embedded in industrial–productivist thinking (Gordon et al. 2021), potentially alienating the very marginalised communities it seeks to include, particularly those with low literacy.

#### Methods, techniques, and processes

The literature demonstrates that the regenerative paradigm meets this criterion. The principle *learn from existing regenerative practices or complementing approaches* and its themes, offer 74 specific elements and 11 case studies. Regeneration scholarship also advocates for *implementing alternative and transformative economic models* like *circular* and *purpose economies*. However, the literature reveals a critical gap in discussing which methods, techniques, and processes are most effective in different situations. As Gordon et al. (2021) note, it remains unclear whether regeneration can challenge the discursive power of industrial–productivist systems if its methods, techniques, and processes remain grounded in the ‘old’ paradigm.

#### Frameworks

The theme *apply regenerative thinking frameworks* reflects this criterion, specifically mentioning *Doughnut Economics*, *Story of Place*, and *Multi-Capitals,* aligning with economic, social, and ecological goals. However, many frameworks are abstract or ambiguous, for example, the *Three Lines of Work* and *Levels of Work* (Mang et al. 2016). While some degree of abstraction can support visionary thinking, the analysed literature lacks clarity on how these frameworks are used. This may present barriers to advancing regenerative thinking and practice. As Hes et al. (2018, p. 2) rightfully ask: “how do we operationalise these abstract concepts?”.

#### Intuitions

The ‘intuitions’ criterion is met through regeneration’s emphasis on inner *awareness* as vital to systems change. Themes like *emotions* and *inner compass* reflect a reevaluation of the role of *intuition* in knowledge transmission (Hardman 2012; Hes and du Plessis 2015). Broadly, the literature lacks clarity on how intuitions can be integrated into dominant systems shaped by linear thinking (Wamsler et al. 2021). Gaps also persist about whether intuitions can coexist with measurements, which some authors critique as a Western construct, raising critical questions about assessing ‘inner change’ in the absence of indicators and metrics. While three articles call for integrating qualitative metrics, they explore this outside the ‘intuition’ criterion.

#### Values

‘Values’ are reflected in the literature through themes of *embracing new leadership* and *developing new values*, supported by *purpose-driven, regenerative*, and *shared leadership* that emphasise collaborative, interdependent *stewardship* roles (Buckton et al. 2023). Values like *equity*, *humility, and being of service* reflect regeneration’s relational and ethical focus. However, the literature overlooks how values are shaped by collective culture and societal beliefs, leading to ambiguity about which—or rather, whose—values should inform regenerative approaches. This raises unresolved questions about which value systems are prioritised, and how to navigate conflicting interpretations of what counts as ‘regenerative’ (Paolini et al. 2024).

#### Metaphysical speculation and theories

These two criteria are grouped, as they are not adequately represented in the literature. Although specific elements like *consciousness* and *spirituality* align with metaphysical speculation, there is an implication that regeneration is ‘the’ paradigm to solve all complex challenges, contrary to the metaphysical notion that paradigms cannot fully explain reality (Masterman 1970). Instead, the literature borrows from Indigenous worldviews to frame broader metaphysical insights. In contrast to the enthusiastic embrace of Indigenous worldviews, the literature often critiques other theocentric worldviews like Judeo-Christian traditions, implying that human–nature separation arose from the idea of man having dominion over nature.

## Discussion

In this section, we interpret our findings through two main lenses. First, we infer how some components of the regenerative paradigm could support a shift away from growth-oriented paradigms in WEIRD societies. We then offer a critical reflection on what we conceptualise as the *Tensions of Paradigm Shifts*: six tensions that may contribute to paradigm blindness in regeneration’s conceptualisation and practice. Italicised text indicates Map components or paradigm criteria, demonstrating a direct connection between our reflections and the findings.

Overall, findings suggest that while the regenerative paradigm has limitations, it fulfils several criteria for what constitutes a paradigm. Hypothetically, it could support a paradigm shift, provided its adoption remains locally grounded, culturally sensitive, protective of community agency, and vigilant against the risk of co-option by dominant global interests. Although our analysis did not directly assess its capacity to replace growth-oriented paradigms, components of the Map indicate that the regenerative paradigm differs significantly from the beliefs Tribe et al. (2015) identified as underpinning neoliberal paradigms.

The regenerative paradigm, thus, could shift competitiveness towards *collaboration*, deregulation towards *participatory and ethical governance,* efficiency towards net-positive benefits, free markets towards *place-based* economies, profit towards *value-based systems* and *multi-capitals frameworks,* consumption towards *sufficiency*, capitalism towards *pluralistic, post-capitalist economic models,* globalisation towards bioregionalism, individualism towards *humans-nature interdependence*, and growth towards *flourishing*. However, the paradigm’s potential to become a "Dominant Social Paradigm" (Dunlap and Van Liere 1978) remains unclear. Moreover, despite its transformative potential, it is vital to critically reflect on the regenerative discourse itself and to maintain the agility and adaptiveness needed to recognise and address any paradigm blindness that may emerge within it.

### Six tensions of paradigm shifts

#### Complexity and the risk of cherry-picking

The regenerative paradigm’s 293 components—7 core principles, 33 themes, and 253 specific elements—reflect its complexity and conceptual diversity, suggesting that the paradigm is a ‘melting pot’ of past ideas, concepts, and worldviews, not all unique to regeneration. Paolini et al. (2024) describe regeneration as a “boundary concept”: a term that bridges diverse fields, facilitating crucial *dialogues* across knowledge silos. Within the paradigm’s complexity, however, definitions can become ambiguous, leading to individuals interpreting regeneration in different ways. This lack of conceptual clarity risks weakening the paradigm, as conflicting interpretations—or selective uptake of components aligning with the current paradigm (e.g. *green infrastructure*)—may dilute its purpose, hinder its uptake, or expose it to co-option.

As a result, policies and initiatives may appear ‘regenerative’ by reflecting the correct *language,* but upon closer scrutiny, they merely pay lip service to the concept. This raises concerns about ‘cherry-picking’ practices that fall short of a paradigm shift. Similar trends occurred with sustainability, where pioneering ideas like Naess’ (1973) were sidelined for ‘pragmatic’ approaches (Peace et al. 2018) such as “sustainable growth”, which Daly (1990) considers an oxymoron. Thus, regeneration risks repeating sustainability’s trajectory if framed into vague slogans like ‘regenerative growth’, undermining the depth needed for a true paradigm shift.

#### Tensions in regenerative practice and systemic constraints

Our findings reveal that current scholarship closely links the regenerative paradigm to practice. Indeed, all core principles and themes are action-oriented. Principles 3 and 7 explicitly focus on regenerative practice, and the paradigm meets all practice-related paradigm criteria. Yet, few sources critically examine how implementation is shaped—and frequently constrained—by institutional structures, power dynamics, and policy realities. This gap reflects a form of paradigm blindness, characterised by an uncritical acceptance of regenerative ideals without addressing the systemic conditions that may hinder their effective translation into practice.

For example, focusing solely on *inner transformation* can divert attention from structural transformations. Indeed, Brownstein et al. (2022) question whether individual actions influence systems transformation because of how difficult systems are to change. They argue, instead, for a symbiosis between individual and structural actions, a focus missing in the literature analysed. Hence, using the Map requires viewing its components through both individual and structural systems lenses. Moreover, while Principle 3 advocates for *practising participatory and ethical governance for regeneration,* challenges exist when integrating this principle in the real world.

Policy failures are widespread in government, reflecting an Intention versus Action systems archetype, where actions do not follow prescribed policy goals (Taveras-Dalmau and Coghlan 2024). Additionally, policymakers are often risk-averse, leading to managerial ineffectiveness (Bozeman and Kingsley 1998). Hence, advancing Principle 3 requires *new mindsets* and embracing *vulnerability, leadership* and *courage*, particularly in the face of system inertia that may prevent the regenerative paradigm from reaching Stage 5: Paradigm Shift and Transcendence (Fig. 1). These challenges may be more pronounced for the regenerative paradigm, as its inclusive ethos contrasts with existing systems that often resist redistribution of power.

Finally, *putting community first* oversimplifies this principle’s complexity. Communities are not homogeneous, making community planning inherently challenging (Sinclair 2008). While Principle 4 *prioritises locals* and their knowledge, we argue that regeneration is not just a question of knowledge, but of power: who holds it and shifts it, both within and across communities. Our analysis also shows that while the regenerative paradigm meets all practice-related criteria* (frameworks*, *tools, models or instruments; methods, techniques, or processes*; and *rules, standards, and judicial decisions)*, barriers such as resource demands, expertise requirements, practical testing, and institutional resistance are seldom addressed. These gaps highlight the need for tangible, data-driven insights to assess the feasibility, applicability, and effectiveness of regenerative practices.

#### Diverse epistemologies, power, and the risk of appropriation

Current knowledge production in regeneration disproportionately aligns with the Global North, perpetuating ownership of what is considered ‘truth’. Questions about who holds power and authority to determine how knowledge, including Indigenous Knowledge, interacts with existing power structures and educational curricula are largely unaddressed. This reflects contested power dynamics, particularly in educational systems, which have historically neglected Indigenous Knowledge systems and even framed those systems as deficient (Fogarty et al. 2018; Wooltorton et al. 2022). Ultimately, all Map components are largely WEIRD societies’ inventions or interpretations borrowed from other cultures or paradigms, often unconsciously.

If such elements arise predominantly from Western contexts, it raises questions about how they can genuinely reflect diverse worldviews or risk inadvertently reproducing new forms of neocolonialism disguised as ‘regeneration’, echoing patterns observed in Western conservation and ‘green economy’ practices (Bobiec et al. 2025). This dynamic is compounded by the technical and abstract nature of regeneration’s language, which risks excluding broader audiences, especially marginalised communities. Language and discourse, like knowledge, hold power. Knowledge cannot be created without a system of information flows in society. Intentionally or not, individuals controlling mastery and access to those discursive resources confer power (Myburgh and Tammaro 2013).

This power/knowledge dynamic (Focault 1972) classifies individuals outside the regeneration community into hierarchical groups of ‘them’ and ‘us’. This asymmetry in power is central to othering, where power depends on the ability of a discourse to impose itself, which depends on the power of those who speak it (Staszak 2020). This othering is compounded by early adopters perceiving regeneration as a superior approach, evident in professional discussions on platforms like LinkedIn.^1^ Regeneration’s widespread adoption requires humble debate, enabling participation and interest from individuals unfamiliar with the concept, not other them.

A final critical reflection also concerns relabelling trends. The focus of many working in systems change has been the protection of biodiversity and commitment to local well-being. Regeneration changes this focus in name only. This renaming risks detracting from humanity’s failure to meet social and ecological goals established as early as the 1970s. Continuously changing terms to, in essence, speak the same language as Indigenous Peoples or early advocates like Naess (1973) does not bring us closer to achieving those goals. While labels matter, they can be counterproductive if they remain merely aspirational, detracting from the urgency of achieving tangible results.

#### Selective use of values and worldviews in regenerative thinking

Regeneration’s emphasis on prioritising *local culture* and *ecological worldviews* introduces tensions. Since values are subjective and culturally specific, defining ‘regenerative outcomes’ is challenging. Little is understood about how values form and shift; hence, a stronger understanding of value change in social-ecological systems is needed (Kendal and Raymond 2019). Finally, understanding how values in WEIRD societies may foster collective hope could be beneficial. Individuals in WEIRD societies also express values related to connection with others and nature, vitality, personal strengths, and spirituality (Harré et al. 2017), which could be leveraged to create regenerative systems outcomes.

Our findings also show that the regenerative paradigm borrows heavily from Indigenous worldviews while overlooking others, raising concerns about appropriation and the need to uplift Indigenous worldviews, rather than repackaging them into a ‘new’ paradigm. While some Indigenous Leaders view regeneration as aligned with their ancient wisdom (Poelina et al. 2022), others like Romero Briones of the First Nations Development Institute criticise it for being “inherited, guarded and perpetuated by white men” (as quoted in Wozniacka 2021). Given that most regeneration scholarship is authored by Western authors, Indigenous Peoples’ inclusion must align with their goals and self-determination. As Gangulu Elder Dr Lilla Watson (2004) states, “If you have come here to help me, you are wasting your time, but if you have come because your liberation is bound up with mine, then let us work together”.

In line with this, regeneration also risks romanticising alternative worldviews, including those of Indigenous Peoples, who sometimes oppose conservation practices and global environmental programmes framed as ‘positive’ by Western institutions (see Mathews 2019; Gaia Amazonas 2023). Moreover, despite scholarship advocating for a shift away from mechanistic thinking, the selective framing of Indigenous traditions as ‘ecocentric’ and Western ones as ‘anthropocentric’ is reductionist in itself. This binary is overly simplistic and risks misrepresenting both. While drawing inspiration from Indigenous worldviews is valuable, it is crucial to recognise that no single worldview, however holistic, is sufficient to drive a global paradigm shift within WEIRD societies.

In parallel, our findings highlight that regeneration discourse tends to ignore or openly critique other worldviews that advocate for similar principles, such as Buddhist or Judeo-Christian theocentric traditions, the latter of which has long emphasised self-restraint, frugality, and stewardship, principles strongly reflected in Pope Francis’ (2015) Encyclical ‘On Care for Our Common Home’, where “a distorted anthropocentrism” is “forcefully criticised” (p. 51). Speaking on behalf of Indigenous worldviews or denying the legitimacy of theocentric traditions risks replicating the very paradigm blindness regeneration seeks to overcome.

#### Interpreting a paradigm not yet fully known

Paradigm shifts often occur at the fringes of dominant systems, which are upheld by entrenched mindsets that resist change or cannot envision alternative ways of thinking (Prince 1994). As Savory (2020) states, “the finest candle-makers in the world could not think of electric lights” because such an idea did not exist within their reality. Advancing the regenerative paradigm without having lived it, while confronting resistance from entrenched systems creates a tension, where individuals are “caught in between different worldviews, not feeling at home in either” (Briciu 2024, p. 2). Hence, any new paradigm—including the regenerative one—is inherently difficult to interpret, as it has not yet been experienced.

#### From disruption to dogma?

Perhaps the most prominent tension of paradigm shifts is that actors’ uncritical acceptance of a ‘new’ paradigm risks becoming an ideology itself. This dynamic is increasingly reflected in scholarly interpretations of the regenerative paradigm, with some authors positioning it as a panacea that can solve all complex social-ecological challenges. For example, Bellato and Pollock (2023, p. 8) state that “a vacuum of ideas is forming that only regenerative thinking can fill”. We argue that no single paradigm holds all the answers, and claiming so perpetuates hegemonic thinking, where everything else is discarded in favour of the paradigm that aligns with our worldview.

The real challenge then lies in striking a balance between adhering to the ‘new’ paradigm so that its adoption is authentic and remaining fluid enough to accept a future alternative. This delicate balance is why, within Meadows’ (2009, p. 164) 12 leverage points, the “power to transcend paradigms” is the deepest. She states:

“*There is yet one leverage point that is even higher than changing a paradigm. That is to keep oneself unattached in the arena of paradigms, to stay flexible, to realise that no paradigm is ‘true’, and that everyone, including the one that sweetly shapes your own worldview, has a tremendously limited understanding of an immense and amazing universe that is far beyond human comprehension”.*

Indirectly, Meadows (2009) speaks to the importance of becoming aware of paradigm blindness and recognising when we are trapped within it. What is needed then is a paradigm dialectic model, like Cronenberg’s (2018) paradigm parley. She argues that a “dichotomous representation of decision-making leaves no room for grey areas…for difference and diversity…for dialogue between two perspectives” (Cronenberg 2018, p. 26). Thus, like Sharpe’s (2013) Three Horizons framework, one must recognise the value of ‘old’ (First Horizon) and ‘new’ (Third Horizon) paradigms, using the Second Horizon to take what works from both into the future.

Such flexibility allows for more creative and effective solutions and new possibilities and innovations unconstrained by existing paradigms, opening individuals to different ways of knowing and fostering their capacity to question and critically evaluate the underlying assumptions of any given paradigm, including the regenerative one. While regeneration should not be absorbed into the old paradigm, with individuals believing they are shifting but remaining in the ‘questioning’ and ‘stretching’ stages (Fig. 1), there is also a risk in proposing regeneration as the *only* solution. Ultimately, achieving this balance depends on our ability to understand the limitations of *all* paradigms, transcending them as needed.

## Theoretical and practical contributions

This study conducted an in-depth analysis of regeneration scholarship, set against a backdrop of what paradigms are and how they form and shift. It identified 14 criteria defining a paradigm, a significant gap in regeneration and broader scholarship. Ultimately, it is unclear whether regeneration constitutes a distinct paradigm, an issue compounded by its nascent nature and the lack of empirical studies using primary data to measure paradigms, paradigm blindness, and whether a paradigm shift has occurred—an important area for future research. The study built on previous literature to conceptualise paradigm blindness as a phenomenon affecting both growth-oriented and regenerative paradigms.

In doing so, we held up a mirror to regeneration scholarship, identifying blind spots and tensions that may reflect paradigm blindness—now or in the future—and hinder its conceptual and practical development, a significant gap in a field often uncritical of itself. Without reflexivity, regeneration risks becoming an ideology that replicates the very paradigm blindness it seeks to overcome. Nonetheless, we share regeneration’s call for a paradigm shift and see regenerative futures as worth pursuing. To this end, our study offers the most comprehensive review of regeneration to date, culminating in a Regenerative Paradigm Map aligned with Kuhn’s (1962, p. 109) framing of paradigms as a vehicle “telling scientists about the entities that nature does or does not contain” and how they behave, providing a “map” that is as essential to science’s continuing development as observation and experimentation.

Masterman (1970, p. 70) interprets Kuhn’s (1962) view as meaning that the “construct sense of paradigms…is the most fundamental one”, guiding scientists in answering the question: “What does a paradigm do?” She argues, “Only with an artefact can you solve a puzzle”. We created the Map as such an artefact, guiding regeneration’s theory and practice, grounded in 320 cross-disciplinary articles. This interdisciplinary foundation ensures the Map generalises regeneration while offering greater richness in conceptualising it, as some principles, themes, or specific elements might have arisen from particular disciplines.

While primarily a conceptual tool, the Map makes complex ideas accessible, adaptable, and actionable by framing principles, themes, and specific elements in action-oriented language. Its interactive version further advances these components through ‘what, why, and how’ pop-ups, guiding interested individuals in WEIRD societies to better understand regeneration, thereby reducing the risk of co-option. Designed to evolve with new insights, the Map maintains relevance as the field advances. Ultimately, this study is an invitation to become aware of one’s paradigm blindness—whether growth-oriented or regenerative—, understand the *Tensions of Paradigm Shifts*, and use the Map to shift from “thinking ourselves into new ways of living” to “living ourselves into new ways of thinking” (Rohr 2003).

## Supplementary Information

Below is the link to the electronic supplementary material.

Supplementary file1 (PDF 647 KB)

## Footnotes

1 An example of such conversations can be seen here.
