# Supplementary material for: From paradigm blindness to paradigm shift? An integrative review and critical analysis of the regenerative paradigm
Source: Ambio. 2025 Sep 12;54(12):1985–2004. doi: 10.1007/s13280-025-02232-7 (PMC12569253; doi:10.1007/s13280-025-02232-7)
Supplement: Supplementary file 1 — Supplementary file1 (PDF 647 KB) [file 13280_2025_2232_MOESM1_ESM.pdf]

Appendix S1. Paradigms stemming from anthropocentrism and ecocentrism. Sourced from Kim et al. (2023), Catton and Dunlap (1978), Colby (1991), Olsen et al. (1992), Milne et al. (2009), Colby (1989), Figueroa-Helland and Raghu (2016), ICT (n.d.), Naess (1973), Rosenhek (2004), Lundmark (2007), and Du Plessis and Brandon (2015).

Belief	Paradigm	Main premise	Underlying beliefs and values
Anthropocentrism	Human exceptionalism	Humans exist independently of nature, making them uniquely different from nonhuman species and disconnected to the natural world.	• Humans are superior to nonhuman beings.<br>• Human societies exist independent from nature.<br>• Human progress can continue without limits.<br>• Humans should have the right to use nature for their own benefit.<br>• Human life is more valuable than nonhuman life.
	Frontier economics	Nature is viewed as infinite, offering endless resources for human use and serving as an unlimited sink for waste by-products. Consequently, the environment is often disregarded, perceived as boundless and irrelevant to the economy.	• Nature is an infinite resource.<br>• Nature can be exploited for human benefit.<br>• Technological solutions will solve any issues arising from nature's exploitation.<br>• Economic growth is the primary goal of development and should be given priority over environmental protection.
	Environmental protection	The frontier economics paradigm began to weaken in the 1960s. The environmental protection paradigm emerged, advocating for 'damage control' that reduced environmental harms. However, it did not improve development or ecological resilience.	• The environment is an economic externality. Command-and-control approaches should be implemented to mitigate these externalities.<br>• Limits to harmful activities should be established.<br>• Environmental agencies are established but do not proactively plan or manage adverse environmental impacts.<br>• Land should be privatised to prevent resource overuse and some set aside for public parks.<br>• Economic growth should be balanced with environmental protection.
	Resource management	Promotes a substantial change in practice that considers multiple forms of capital and recognises economic dependence on ecological systems.	• Economic and ecological goals should be integrated into development.<br>• Efficient resource use is needed to optimise the balance between consumption and conservation.<br>• Resources are intended for human use, but their management should consider their multiple, often overlapping values.<br>• Population levels should be stabilised.<br>• New initiatives to manage the global commons should be established.
	Sustainable development	Promotes a balanced approach that integrates social, economic, and environmental goals, aiming for long-term stability, resource durability, and precautionary measures.	• Modern industrial societies are seriously disturbing the balance of nature.<br>• Science and technology have improved our quality of life, freedom and independence.<br>• Economic stability should be the major goal of government.<br>• Actions should not be taken unless their long-term consequences are clear.
Ecocentrism	Indigenous	An alternative to WEIRD societies, with a worldview grounded in decolonial, non-anthropocentric principles that considers Earth as a living being sharing an interdependent and spiritual relationship with humanity. This paradigm existed long	• Decolonial approaches should be pursued.<br>• Humans have a spiritual connection to nature.<br>• People, objects, and nature are all interconnected, with law, kindship and spirituality reinforcing this connectedness.<br>• The land is sacred, and humans should live in balance with Mother Earth.<br>• Time is non-linear and cyclical.

		before anthropocentrism, but colonialism and imperialism's cultural genocide suppressed it.	
	Deep ecology	Advocates for a profound shift in human consciousness and values, recognising all life has intrinsic value, regardless of their utility to humans. Deep ecology proposes a "relational, total-field image" where humans are integral components of Earth's ecosystem, and all life is interdependent.	• The equal right to live and flourish is applicable to all forms of life, not just humans.<br>• All beings are interconnected, and their existence is defined by their relationships with others.<br>• Humans should live in ecological harmony and balance with nature.<br>• Local autonomy and decentralisation should be pursued.<br>• Humans need a radical shift in consciousness to reconnect with Earth through spiritual awareness.
	Eco-development	Seeks to restructure society's relationship with nature to be synergistic, mimicking ecosystem processes. It replaces the closed economic model with a biophysical model embedded in nature, integrating ecological preservation with social equity and cultural values.	• Human activities should be synergistic with ecosystem processes and services.<br>• Social, ecological, and economic concerns need to be better reorientated and integrated.<br>• Economies should be restructured according to ecological principles to prevent pollution.<br>• Humans should move beyond efficiencies to design economies that mimic nature.<br>• Ecological uncertainty needs to be incorporated into economic modelling and planning.<br>• People do not have a right to continue with business as usual.
	New ecological paradigm	The world is an interdependent and interconnected living system where humans and nature are partners in the process of co-creation and co-evolution. The world is dynamic and ever-changing driven by interactions of its elements.	• The earth is like a spaceship with limited room and resources.<br>• All life is interconnected within a single, complex living system.<br>• Humanity must reconcile our relationship with nature, aligning activities with ecological systems to enable mutually supportive co-evolution and symbiosis.<br>• Whole living systems thinking needs to replace mechanistic thinking.<br>• Humans must embrace approaches that enhance the capacity of socio-ecological systems to self-organise and evolve.

Appendix S2. Assessment of regenerative paradigm's components against the 14 paradigm criteria of the analytical framework.

Paradigm Criteria	Criteria Description	Regenerative Paradigm Map Components & Connection to Paradigm Criterion		
		**Core Principles**	**Themes**	**Specific Elements**
Principle	Fundamental, guiding ideas that govern perception and action within a paradigm, providing an overarching structure for how the paradigm operates and evolves.	Start with inner transformation<br>Adopt pluralistic, post-capitalist economic models<br>Practice participatory and ethical governance for regeneration<br>Put community first<br>Work with place as living<br>Use systems approaches<br>Learn from existing regenerative practices or complementary approaches		
Rules, Standards & Judicial Decisions	Formalised or preconceived norms that paradigm holders adhere to, with their consensus acting as prerequisites for how the paradigm operates, defining boundaries, procedures, and acceptable practices.		Prioritise locals	Collaboration<br>Community engagement<br>Local culture<br>Green Infrastructure<br>Participatory<br>Ethics<br>De-growth
Epistemology & Knowledge	Concerns the structure, nature, and evolution of knowledge within a paradigm. This includes what we accept as 'knowledge', how it is vindicated by evidence and how it shifts and grows.		Elevate your consciousness<br>Integrate diverse knowledge<br>Embrace a new mindset<br>Develop multi-literacy<br>Develop soft skills<br>Include diverse perspectives<br>Consider systems properties	Wisdom<br>Ecological knowledge<br>Integrated intelligence<br>Learning<br>Metacognition<br>Alternative thinking<br>Being and function thinking<br>Complexity thinking<br>Innovative thinking<br>Integral thinking<br>Outside-in thinking<br>Eco-Literacy<br>Green Service Literacy<br>Pattern Literacy<br>Psychological Literacy<br>Regenerative Literacy<br>Social Literacy<br>Feminist Perspectives<br>World-Centric Perspective
Beliefs & Assumptions	Relates to the cultural and ideological underpinnings of a paradigm that dictate the specific ideas individuals have about aspects of life that they are convinced are		Catalyse community empowerment	Biocentrism<br>Co-equal partnership<br>Co-evolution

	true regardless of potentially contrary evidence. These are often unspoken or implicit but profoundly shape the paradigm's development and application.			Human-nature interdependence<br>Learn from & work with nature<br>Nature As Stakeholder<br>Intrinsic Value of Life<br>Awareness<br>Caring<br>Connection<br>Mutuality<br>Reciprocity<br>Harmony<br>Rooted Nurture<br>Solidarity<br>Fellowship<br>Stewardship<br>Holistic & Genuine Wealth<br>Sufficiency<br>Value-Based System<br>Integration<br>Right Relationship<br>Collective action<br>Trust & Group Identity<br>Shared vision and purpose
Ontology & Worldviews	Refers to the foundational perspectives and assumptions about reality that underpin a paradigm, including subconscious ways of seeing and what we accept as 'real'.		Shift to an ecological worldview<br>Nourish people and place connectedness<br>Move beyond capitalism	Monetary Transition<br>Decolonial Approaches<br>Indigenous Culture<br>Indigenous Knowledge<br>Indigenous Law<br>Indigenous Science<br>Indigenous Values<br>Indigenous Worldviews<br>Resident economy<br>Essence<br>Patterns<br>Place-Sourced Potential<br>Uniqueness<br>Vocation<br>Paradoxes of Place<br>Sense Making<br>Complexity<br>Emergence<br>Flows<br>Leverage<br>Nested Systems<br>Networks<br>Relationships<br>Self-Organising<br>System Purpose<br>Living Systems<br>Socio-Ecological Systems

				System Dynamics<br>Whole Systems
Political Institutions	Structures of governance, power, and decision-making that influence and regulate a paradigm. They shape its application through policy and societal organisation.		Design Inclusive Policy Frameworks<br>Re-Design Governance Structures<br>Include Indigenous People	Collaborative & Diverse Governance<br>Partnerships<br>Policy Action & Reform<br>Socio-Political Reconnection<br>Sociocracy & Holacracy<br>Transparent Politics<br>Community of Practice<br>Agency<br>Bottom-Up Over Top-Down
Tools, Models or Instruments	Conceptual, instrumental, or analytical tools supplied by the paradigm itself, which are employed consciously or unconsciously to apply or operationalise the paradigm, evolving only when they no longer address anomalies within the paradigm.		Apply regenerative tools for design & evaluation Get Inspired with practical, regenerative case studies<br>Use regenerative and place-based metrics	Generative Questions<br>Guilds<br>Indicators & Metrics<br>Measuring Flourishing<br>Bullit Centre<br>Ecovida Network<br>Fogo Island Inn<br>Green Building, Switzerland<br>Las Salinas Project, Vina del Mar, Chile<br>Loreto Bay<br>Lyle Centre For Regenerative Studies<br>Playa Viva, Juluchuca, Mexico<br>Soneva Resorts<br>Torres del Paine Legacy Fund<br>Visit Flanders<br>French EcoQuartier<br>LENSES<br>Living Building Challenge & Living Community Challenge<br>MEFA<br>One Planet Communities<br>REGEN<br>Regenerative Capacity Index<br>Regenerative Design Evaluation Tool<br>Regenerative Design Games<br>Regenerative Development Evaluation<br>Technology & Eco-Innovations<br>WELL Building Standard<br>Wilderness-Based Checklist
Language	Refers to the terminology and shared system of communication that shapes methodologies, unites practitioners, expresses the paradigm's ideas, and evolves alongside theoretical and methodological shifts		Embrace a new discourse	Narrative<br>Stories<br>Voice<br>New Language

	to maintain a common base for inquiry and collaboration.			
Methods, Techniques or Processes	General methodological prescriptions or standard ways of applying the paradigm’s rules, acting as instrumental techniques that bring those rules into the real world to achieve its objectives. They provide actionable pathways for implementation.		Transform practice to be dynamic, relational, and aligned with living systems<br>implement alternative and transformative economic models<br>Use systems thinking<br>Activate placemaking<br>Consider place attributes<br>Integrate complementary approaches, concepts, & disciplines<br>Co-create through regenerative, adaptive, iterative processes Apply ecological and systemic design methods	Circularity<br>Design for Evolution<br>Circular Economy<br>Creative Economy<br>Diverse & Inclusive Business Models<br>Generosity Economy<br>Living Organisations<br>Purpose Economy<br>Regenerative Economy<br>Sacred Economy<br>Sharing Economy<br>Social Enterprise<br>More Than'<br>Listening<br>Co-Creation & Co-Design<br>Multi-stakeholder dialogue<br>Backcasting<br>Ecological Performance Standards<br>Ecosystem Services Analysis<br>Genius of Place<br>Life's Principles<br>Theory U<br>Human-Centred<br>Collaborative Dialogue of Discovery<br>Design Thinking<br>Generative Questions<br>Iteration<br>Monitoring<br>Evolving Roles<br>Regenerative Design Process<br>SmartMode<br>Sustainability Life Cycle Assessment<br>Alternative Tourism<br>Bio-Inspired Design<br>Bioconnectivity<br>Biomimicry<br>Biophilia<br>Conscious Marketing<br>Conscious Travel<br>Creative Tourism<br>Mutualism<br>Permaculture<br>Positive Development

				Positive Psychology<br>Regenerative Action<br>Regenerative Agriculture<br>Regenerative Architecture<br>Regenerative Community Development<br>Regenerative Compatibility<br>Regenerative Development & Design<br>Regenerative Ecologies<br>Regenerative Economics<br>Regenerative Education<br>Regenerative Experiences<br>Regenerative Futures<br>Regenerative Living Landscape Development<br>Regenerative Sustainability<br>Regenerative Urbanism & Built Environment<br>Smart Ecology<br>Transformational Travel
Frameworks	Structured conceptual systems that guide understanding, observation, and action within paradigms, framing scientific progress and the evolution of knowledge.		Apply regenerative thinking frameworks	Multi-Capitals<br>Visitor-Host-Place<br>Story of Place<br>Action Framework for Regenerative Business<br>CityCraft<br>Decision-Making Framework for Regenerative Practices<br>Doughnut Economics<br>Ecological Wisdom Inspired Planning Support Systems<br>Law of Three<br>Levels of Work<br>One Planet Living<br>Perkins+Will Framework<br>Regen Concept Framework<br>Regenerative Decision-Making<br>Regenerative Development Goals<br>Regenerative Design Framework<br>Regenerative Tetrad<br>Three Lines of Work<br>Framework thinking<br>Indigenous Frameworks
Intuitions	Intuitions are shared, often tacitly communicated instincts and insights within a paradigm, shaping its unspoken foundations beyond its formalised rules. These often emerge from personal or collective experience and can shape decision-making.		Honour negative emotions<br>Channel positive emotions	Denial<br>Despondency<br>Grief<br>Guilt<br>Shame

			Acknowledge your inner compass	Courage<br>Hope<br>Optimism<br>Intuition<br>Presencing<br>Positivity
Values	The shared ethical, moral, and social principles that underpin and justify the paradigm, expressing collective judgments about what is desirable and guiding actions by defining how things ought to be in alignment with the paradigm's beliefs, thus influencing broader paradigm goals.		Embrace new leadership<br>Develop new values	Purpose-Driven Leadership<br>Regenerative Leadership<br>Shared Leadership<br>Sustainability Leadership<br>Transformational Leadership<br>Being Of Service<br>Equity<br>Generosity<br>Humility<br>Inclusivity<br>Integrity<br>Justice<br>Respect<br>Purpose<br>Responsibility<br>Transparency<br>Empathy<br>Vulnerability
Metaphysical Speculation	The foundational assumptions and philosophical interpretations that shape how phenomena are described and understood, influencing ideas about existence and reality. Metaphysical speculation acknowledges that paradigms, while unifying perspectives, never fully explain all observable facts.			Consciousness<br>Spirituality
Theories	Structured body of knowledge derived from scientific observations and experiments that serves as foundational framework within a paradigm to explain phenomena and make predictions, bridging empirical evidence with broader scientific understanding.		Systems theory (not explicitly in map but informed by the 'Use systems approaches' principle.	Topophilia

Appendix S3. PRISMA 2020 Checklist for integrative review.

Topic	No.	Item	Location where item is reported
**TITLE**			
**Title**	1	Identify the report as a systematic review.	Section 3.2, page 8
**ABSTRACT**			
**Abstract**	2	See the PRISMA 2020 for Abstracts checklist	
**INTRODUCTION**			
**Rationale**	3	Describe the rationale for the review in the context of existing knowledge.	Section 1, Lines 46 - 63, page 2
**Objectives**	4	Provide an explicit statement of the objective(s) or question(s) the review addresses.	Section 1, Lines 65 - 74, page 3
**METHODS**			
**Eligibility criteria**	5	Specify the inclusion and exclusion criteria for the review and how studies were grouped for the syntheses.	Section 3.3, Lines 279 - 281 and 286 - 290, page 11
**Information sources**	6	Specify all databases, registers, websites, organisations, reference lists and other sources searched or consulted to identify studies. Specify the date when each source was last searched or consulted.	Section 3.3, Lines 273 - 290, page 11
**Search strategy**	7	Present the full search strategies for all databases, registers and websites, including any filters and limits used.	Section 3.3, Lines 273 - 290, page 11
**Selection process**	8	Specify the methods used to decide whether a study met the inclusion criteria of the review, including how many reviewers screened each record and each report retrieved, whether they worked independently, and if applicable, details of automation tools used in the process.	Section 3.3, Lines 283 - 291, page 11
**Data collection process**	9	Specify the methods used to collect data from reports, including how many reviewers collected data from each report, whether they worked independently, any processes for obtaining or confirming data from study investigators, and if applicable, details of automation tools used in the process.	Section 3.3, Figure 1, page 12
**Data items**	10a	List and define all outcomes for which data were sought. Specify whether all results that were compatible with each outcome domain in each study were sought (e.g. for all measures, time points, analyses), and if not, the methods used to decide which results to collect.	N/A

Topic	No.	Item	Location where item is reported
	10b	List and define all other variables for which data were sought (e.g. participant and intervention characteristics, funding sources). Describe any assumptions made about any missing or unclear information.	N/A
**Study risk of bias assessment**	11	Specify the methods used to assess risk of bias in the included studies, including details of the tool(s) used, how many reviewers assessed each study and whether they worked independently, and if applicable, details of automation tools used in the process.	Section 3.3, Figure 1, page 12
**Effect measures**	12	Specify for each outcome the effect measure(s) (e.g. risk ratio, mean difference) used in the synthesis or presentation of results.	N/A
**Synthesis methods**	13a	Describe the processes used to decide which studies were eligible for each synthesis (e.g. tabulating the study intervention characteristics and comparing against the planned groups for each synthesis (item 5)).	Section 3.4, Lines 323 - 330, page 13
	13b	Describe any methods required to prepare the data for presentation or synthesis, such as handling of missing summary statistics, or data conversions.	N/A
	13c	Describe any methods used to tabulate or visually display results of individual studies and syntheses.	Section 3.4, Lines 333 - 338, page 13
	13d	Describe any methods used to synthesize results and provide a rationale for the choice(s). If meta-analysis was performed, describe the model(s), method(s) to identify the presence and extent of statistical heterogeneity, and software package(s) used.	N/A
	13e	Describe any methods used to explore possible causes of heterogeneity among study results (e.g. subgroup analysis, meta-regression).	N/A
	13f	Describe any sensitivity analyses conducted to assess robustness of the synthesized results.	N/A
**Reporting bias assessment**	14	Describe any methods used to assess risk of bias due to missing results in a synthesis (arising from reporting biases).	Section 3.5, Lines 346 - 354, Page 14
**Certainty assessment**	15	Describe any methods used to assess certainty (or confidence) in the body of evidence for an outcome.	N/A
**RESULTS**			

Topic	No.	Item	Location where item is reported
**Study selection**	16a	Describe the results of the search and selection process, from the number of records identified in the search to the number of studies included in the review, ideally using a flow diagram.	Section 3.3, Figure 1, page 12
	16b	Cite studies that might appear to meet the inclusion criteria, but which were excluded, and explain why they were excluded.	Section 3.3, Figure 1, page 12
**Study characteristics**	17	Cite each included study and present its characteristics.	N/A. Too many studies to cite.
**Risk of bias in studies**	18	Present assessments of risk of bias for each included study.	Section 3.3, Figure 1, page 12
**Results of individual studies**	19	For all outcomes, present, for each study: (a) summary statistics for each group (where appropriate) and (b) an effect estimate and its precision (e.g. confidence/credible interval), ideally using structured tables or plots.	N/A. Thematic analysis conducted.
**Results of syntheses**	20a	For each synthesis, briefly summarise the characteristics and risk of bias among contributing studies.	N/A. Thematic analysis conducted.
	20b	Present results of all statistical syntheses conducted. If meta-analysis was done, present for each the summary estimate and its precision (e.g. confidence/credible interval) and measures of statistical heterogeneity. If comparing groups, describe the direction of the effect.	N/A. No statistical syntheses conducted.
	20c	Present results of all investigations of possible causes of heterogeneity among study results.	N/A. Thematic analysis conducted.
	20d	Present results of all sensitivity analyses conducted to assess the robustness of the synthesized results.	N/A. No sensitivity analyses conducted.
**Reporting biases**	21	Present assessments of risk of bias due to missing results (arising from reporting biases) for each synthesis assessed.	Section 3.5, Lines 346 - 354, page 14
**Certainty of evidence**	22	Present assessments of certainty (or confidence) in the body of evidence for each outcome assessed.	N/A. Thematic analysis conducted.
**DISCUSSION**			

Topic	No.	Item	Location where item is reported
**Discussion**	23a	Provide a general interpretation of the results in the context of other evidence.	Sections 4 and 5.
	23b	Discuss any limitations of the evidence included in the review.	Section 3.5, Lines 346 - 354, page 14
	23c	Discuss any limitations of the review processes used.	Section 3.5, Lines 346 - 354, page 14
	23d	Discuss implications of the results for practice, policy, and future research.	Section 6, pages 34 - 35
**OTHER INFORMATION**			
**Registration and protocol**	24a	Provide registration information for the review, including register name and registration number, or state that the review was not registered.	Review was not registered.
	24b	Indicate where the review protocol can be accessed, or state that a protocol was not prepared.	Protocol was not prepared.
	24c	Describe and explain any amendments to information provided at registration or in the protocol.	N/A
**Support**	25	Describe sources of financial or non-financial support for the review, and the role of the funders or sponsors in the review.	No funding provided.
**Competing interests**	26	Declare any competing interests of review authors.	No competing interests to declare.
**Availability of data, code and other materials**	27	Report which of the following are publicly available and where they can be found: template data collection forms; data extracted from included studies; data used for all analyses; analytic code; any other materials used in the review.	Any data can be requested from the authors.

PRISMA Abstract Checklist

Topic	No.	Item	Reported?
**TITLE**			
**Title**	1	Identify the report as a systematic review.	Yes
**BACKGROUND**			
**Objectives**	2	Provide an explicit statement of the main objective(s) or question(s) the review addresses.	Yes
**METHODS**			
**Eligibility criteria**	3	Specify the inclusion and exclusion criteria for the review.	No
**Information sources**	4	Specify the information sources (e.g. databases, registers) used to identify studies and the date when each was last searched.	No
**Risk of bias**	5	Specify the methods used to assess risk of bias in the included studies.	No
**Synthesis of results**	6	Specify the methods used to present and synthesize results.	Yes
**RESULTS**			
**Included studies**	7	Give the total number of included studies and participants and summarise relevant characteristics of studies.	Yes
**Synthesis of results**	8	Present results for main outcomes, preferably indicating the number of included studies and participants for each. If meta-analysis was done, report the summary estimate and confidence/credible interval. If comparing groups, indicate the direction of the effect (i.e. which group is favoured).	No
**DISCUSSION**			
**Limitations of evidence**	9	Provide a brief summary of the limitations of the evidence included in the review (e.g. study risk of bias, inconsistency and imprecision).	No
**Interpretation**	10	Provide a general interpretation of the results and important implications.	Yes
**OTHER**			
**Funding**	11	Specify the primary source of funding for the review.	No
**Registration**	12	Provide the register name and registration number.	No

From: Page MJ, McKenzie JE, Bossuyt PM, Boutron I, Hoffmann TC, Mulrow CD, et al. The PRISMA 2020 statement: an updated guideline for reporting systematic reviews. MetaArXiv. 2020, September 14. DOI: 10.31222/osf.io/v7gm2. For more information, visit: www.prisma-statement.org.

Cited References

Catton, W., & Dunlap, R. E. (1978). Environmental Sociology: A New Paradigm. *The american sociologist*, 41-49.

Colby, M. (1989). *The Evolution of Paradigms of Environmental Management in Development*. The World Bank

Colby, M. (1991). Environmental Management in Development: The Evolution of Paradigms. *Ecological Economics*, *3*, 193-213.

Du Plessis, C., & Brandon, P. (2015). An Ecological Worldview as Basis for a Regenerative Sustainability Paradigm for the Built Environment. *Journal of Cleaner Production*, *109*, 53-61. https://doi.org/10.1016/j.jclepro.2014.09.098

Figueroa-Helland, L., & Raghu, P. (2016). Indegeneity Vs "Civilization": Indigenous Alternatives to the Planetary Ecological Rift. In J. Smith, M. Goodhart, P. Manning, & J. Markoff (Eds.), *Social Movements and World-System Transformation*. Routledge.

ICT. (n.d.). *Indigenous Worldviews Vs Western Worldviews*. Indigenous Corporate Training. Retrieved 4 Sept 2024 from https://www.ictinc.ca/blog/indigenous-worldviews-vs-western-worldviews

Kim, J. J. H., Betz, N., Helmuth, B., & Coley, J. D. (2023). Conceptualizing Human–Nature Relationships: Implications of Human Exceptionalist Thinking for Sustainability and Conservation. *Topics in Cognitive Science*, *15*(3), 357-387. https://doi.org/10.1111/tops.12653

Lundmark, C. (2007). The New Ecological Paradigm Revisited: Anchoring the Nep Scale in Environmental Ethics. *Environmental Education Research*, *13*(3), 329-347. https://doi.org/10.1080/13504620701430448

Milne, M. J., Tregidga, H., & Walton, S. (2009). Words Not Actions! The Ideological Role of Sustainable Development Reporting. *Accounting, Auditing & Accountability Journal*, *22*(8), 1211-1257. https://doi.org/10.1108/09513570910999292

Naess, A. (1973). The Shallow and the Deep, Long-Range Ecology Movement. A Summary. *Inquiry*, *16*(1-4), 95-100. https://doi.org/10.1080/00201747308601682

Olsen, M., Lodwick, D., & Dunlap, R. (1992). *Viewing the World Ecologically*. USA. Westview Press.

Rosenhek, R. (2004). Deep Ecology: A Radical Transformation of Consciousness. *Biodiversity*, *5*(4), 45-46. https://doi.org/10.1080/14888386.2004.9712749
